# Distal spread and tumor regression patterns following preoperative chemoradiotherapy in rectal cancer patients

**DOI:** 10.3906/sag-2007-102

**Published:** 2021-09-07

**Authors:** İlter ÖZER, Neslihan İnci ZENGİN, Hacı Murat ÇAYCI, Adem YÜKSEL, Tahsin DALGIÇ, Murat ULAŞ, Erdal Birol BOSTANCI, Musa AKOĞLU

**Affiliations:** 1 Department of Gastroenterological Surgery, Eskişehir Osmangazi University, Faculty of Medicine, Eskişehir, Turkey; 2 Department of Pathology, Minister of Health Ankara City Hospital, Ankara, Turkey; 3 Department of Gastroenterological Surgery, Bursa Şevket Yılmaz Training and Research Hospital, Bursa, Turkey; 4 Department of Gastroenterological Surgery, Kocaeli Derince Training and Research Hospital, Kocaeli, Turkey; 5 Department of Gastroenterological Surgery, Minister of Health Ankara City Hospital, Ankara, Turkey

**Keywords:** Rectum cancer, neoadjuvant therapy, resection margin

## Abstract

**Background/aim:**

This study aimed to evaluate the regression pattern with the distal intramural spread (DIS) of rectum cancer after preoperative chemoradiation.

**Materials and methods:**

Specimens from 56 patients who underwent radical resection after preoperative chemoradiation for rectal cancer were examined. The regression pattern (total, fragmented) of the tumor was recorded. DIS status was evaluated by creating sections 0.2 to 0.3 cm thick.

**Results:**

A single macroscopic residual area was detected in all specimens. In 10 patients (17.8 %), pathologically complete responses were identified, and DIS was detected in 33 patients (58.9%). The average DIS distance was 0.56 ± 0.3 cm (range 0.2 – 1.8 cm); the spread was < 1 cm in 87.9% of the patients (29/33). The overall survival rates for 5 and 7 years were 76.8% and 73.2%, respectively. The survival rates between patients with and without DIS were not statistically different (94.6 ± 5.5 vs. 75.1 ± 10.2 months, respectively). In all of the patients, tumor regression pattern was total shrinkage of the tumor.

**Conclusion:**

A sufficient distal resection margin for rectal cancer after preoperative chemoradiation is 1 cm in the vast majority of cases. However, DIS may exceed 1 cm in a small proportion of patients.

## 1. Introduction

Neoadjuvant treatment in the form of chemoradiation has been widely used for rectal cancer, and preoperative chemoradiation is currently regarded as the standard approach for patients with locally advanced or lymph node involvement rectal cancer [1–3]. Preoperative chemoradiation may lead to marked tumor regression in a considerable proportion of patients. Moreover, in some patients, complete responses may be seen [1–3]. However, the microscopic extension of the tumor after regression of the gross tumor has not been sufficiently demonstrated.

A 2-cm distal surgical margin is generally accepted in rectal cancer patients, although this widely depends on information obtained from rectal cancer patients without neoadjuvant chemoradiotherapy (NCRT) [4,5]. However, more recently, a distal resection margin ≤ 1 cm has also been found to be acceptable in patients receiving NCRT [6,7]. This issue has been examined in a limited number of studies. The extent of microscopic intramural spread beyond the gross margin of the tumor should be thoroughly investigated to obtain a clear distal margin in patients receiving NCRT. There is, however, limited information about the pattern of regression in the literature. Both in regression with total shrinkage of the tumor [1] and with a fragmented pattern, leaving residual clusters of cancer cells distant from the gross tumor [8] has been suggested, in only a few studies and with relatively low numbers of patients.

This study aims to evaluate the extent of distal microscopic spread and the regression pattern after NCRT in rectal cancer patients.

## 2. Materials and methods

The study was approved by the Institutional Review Board. 2011 and 2013, the specimens of rectal cancer patients who underwent radical surgery after NCRT were prospectively evaluated. The patients were evaluated by physical examination, routine laboratory tests, colonoscopy, colonoscopic biopsy, and computerized tomography. The distance of the distal tumor margin from anal verge was determined preoperatively by rectoscopic exam. Magnetic resonance imaging, endosonography, and positron emission tomography (PET) were performed when necessary. The indications for NCRT were clinical T3 and T4 tumors and/or suspected lymph node metastasis. All patients included in the study received NCRT. Radiotherapy (4500–5040 cGy) was administered in combination with 5-fluorouracil in all patients. The patients underwent resection with mesorectal excision approximately for 6 to 10 weeks after NCRT. Since some of the patients were evaluated in other centers before neoadjuvant treatment, some data about pretreatment could not be obtained. Patients with distant metastasis were excluded. Age, sex, localization of the tumor, time of surgery after NCRT, distance to surgical margin, number of lymph nodes, lymphatic invasion, T stage, N status, type of operation, distal intramural spread (DIS), status of distal and circumferential surgical margins, and long-term survival were recorded.

Histopathologic evaluation was performed by a single pathologist. The entire distance between the distal margin of the tumor and the distal surgical margin was totally mapped, the whole rectal wall was evaluated using 0.2–0.3 cm thick macroscopic sections, and the extent of distal spread and pattern of regression were recorded. Size, localization, distance to the distal surgical margin, status of circumferential resection (radial) margin (CRM), and tumor regression grade (TRG) were also recorded. Staging was performed according to the 7th AJCC classification [9]. TRG was evaluated and graded according to a modified version of Ryan’s regression grading system [9,10] (see Table 1).

A macroscopic margin was defined as the distance between the distal edge of the macroscopic tumor (lesion or scar tissue) and the distal surgical margin. A microscopic margin was defined as the distance between the most distally located tumor cells and the distal surgical margin. DIS was defined as the distance between the edge of the macroscopic tumor (lesion or scar) and the most distally located tumor cells. Pathological complete response was defined as the absence of tumor cells in the specimen.

Follow-up information was obtained from hospital records and telephone interviews.

### 2.1. Statistical analyses

Statistical analyses were performed using SPSS 20.0 (IBM Corp., Armonk, NY, USA). Numerical variables are expressed as mean ± standard deviation (SD), and categorical variables are given as frequencies. Survival analyses were performed by Kaplan–Meier and Log-rank tests. In the test of two-sided hypotheses, a p-value < 0.05 was considered as statistically significant.

## 3. Results

Of the 56 patients included in the study, 47 underwent low anterior resection (LAR), and 9 patients underwent abdominoperineal resection (APR). None of the patients had distant metastasis at the time of surgery. The clinicopathological characteristics of the patients are shown in Table 2. According to the preoperative evaluation, the mean distance of the tumor from the anal verge was 6.46 ± 2.6 (2–12) cm. Ten patients (17.9%) showed pathological complete response according to the pathological evaluation of the resected specimen. In all the patients, including those with complete response, an inflammatory and fibrous residual lesion was observed.

### 3.1. Distal intramural spread status

The distal surgical margin was free of tumor cells in all patients. DIS was less than 1 cm, except in four patients: two had 1 cm DIS, the other two had 1.2 and 1.8 cm DIS. Among patients without a complete response (46 patients), DIS was not observed in 13 patients (28.3%). In 22 patients (47.8%) ≤ 5 mm, DIS was seen, and the remaining seven patients showed 6–9 mm DIS.

### 3.2. Circumferential radial margin status

The mean distance to CRM was 0.91 ± 0.76 cm when patients with complete responses were excluded. CRM was positive in three patients. In two patients, the distance to CRM was ≤ 1 mm.

### 3.3. Lymph node status

The mean number of harvested lymph nodes was 26.83 ± 8.91 (range 12–46). Of the 56 patients, 17 had metastatic lymph nodes and the remaining 39 were N0. All histopathological outcomes are summarized in Table 3.

### 3.4. Overall survival

**Figure 1:**
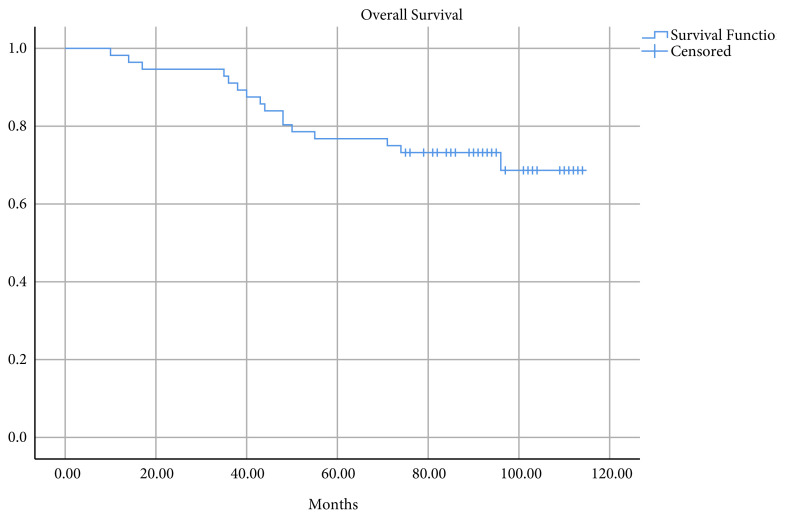
Kaplan–Meier curves of overall survival in the cohort.

**Figure 2:**
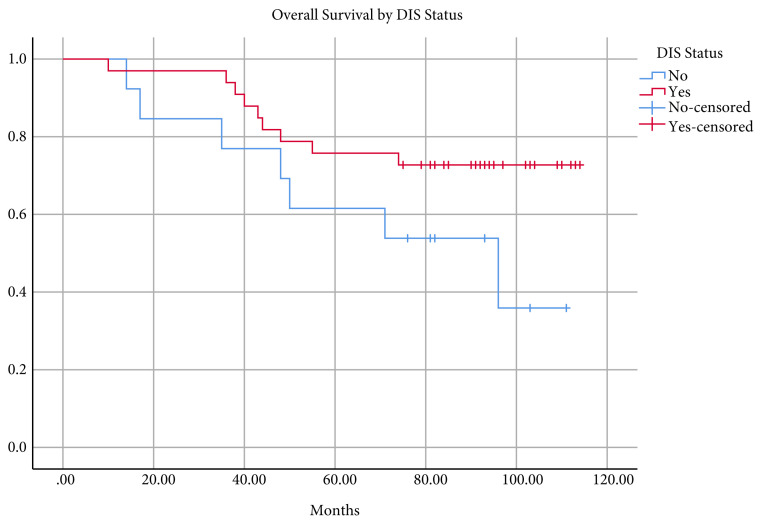
Kaplan-Meier curves of overall survival by DIS status in the cohort (p > 0.05).

**Figure 3:**
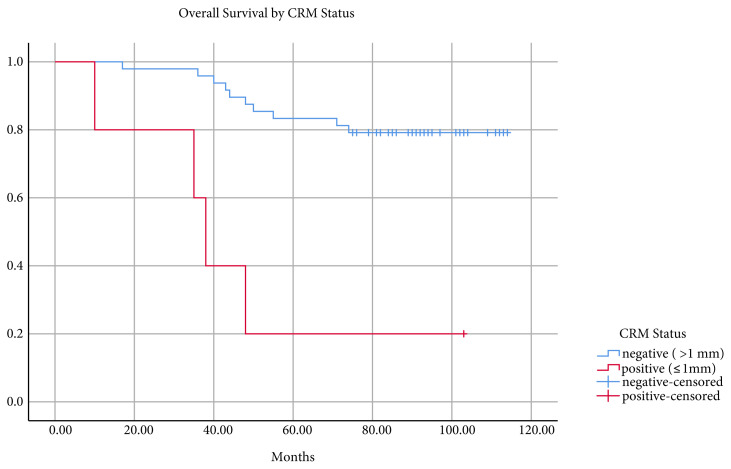
Kaplan–Meier curves of overall survival by CRM status in the cohort (p < 0.001).

**Figure 4:**
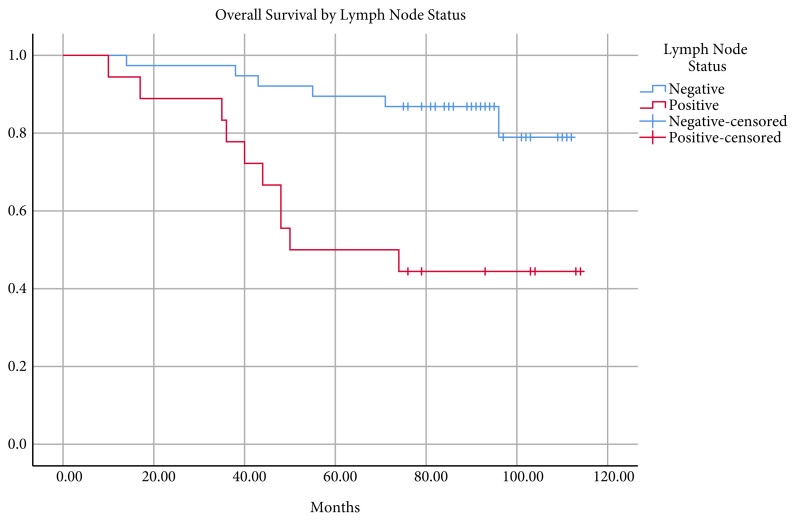
Kaplan–Meier curves of overall survival by lymph node status in the cohort (p < 0.05).

The mean follow-up was 79.4 ± 26.6 (range 10–114) months, and the overall survival was 93.8 ± 4.5 (95% confidence interval 85–102.5) months. The five- and seven-year survival rates were 76.8% and 73.2%, respectively (Figure 1). Sixteen deaths occurred during follow-up. Of these, nine (56.2%) had DIS, while seven did not. Survival was not statistically different between patients with and without DIS (Figure 2). In patients with CRM distance ≤ 1 mm, mean survival was 46.8 ± 13.7 (95% confidence interval 19.8–73.6) and 100.2 ± 4.0 (95% confidence interval 92.3–108) months in patients with CRM distance > 1mm. The difference was statistically significant (p < 0.001) (Figure 3). The mean survival by lymph node status (N+, N0) was 72.9 ± 9.7 (95% confidence interval 53.9–91.9) months and 101.1 ± 3.9 (95% confidence interval 93.3–108.8) months, respectively. The difference was statistically significant (p < 0.05) (Figure 4).

## 4. Discussion

In addition to the histopathological evaluation of the distal surgical margin, we made a detailed study of the entire rectal wall, from the macroscopic tumor (or macroscopic residual lesion) to the distal surgical margin after NCRT. This area was mapped with 0.2–0.3 cm thick macroscopic sections, smaller than those used in previous studies, to thoroughly evaluate the entire rectal wall distal to the tumor. Although there are studies concerning the distal spread of rectal cancer, there has, to date, been a lack of studies performing such a detailed histopathological evaluation on distal spread and regression patterns.

However, in 2005, Mezhir et al. [1] evaluated DIS after preoperative therapy on 20 patients. The pathological evaluation of the distal spread was performed using 0.5 cm cuts. The extent of the spread was 1 cm in five patients (25%); in only one patient was the distal spread more than 1 cm, although the specific spread was 2.5 cm. The authors concluded that, in patients having received preoperative therapy, a 2 cm distal margin was adequate, although they also stated that intramural spread rarely exceeded 1 cm from the gross margin [1]. Our study included 56 patients on whom histopathological evaluation was conducted using 0.2–0.3 cm cuts macroscopically. The distance from the gross lesion margin and the surgical margin was evaluated similarly. In our study group, ≥ 1 cm DIS was observed only in four patients (7.1%). One patient, with a pT3N2, poorly differentiated tumor with perineural and lymphovascular invasion, showed 1.8 cm DIS. Extensive DIS has been shown to be associated with poor histopathological features, advanced disease, and poor survival rates in non-irradiated rectal cancers [11,12], consistent with our findings. Of our patients, one with 1.2 cm DIS and two with 1 cm DIS had metastatic lymph nodes as well as perineural and/or lymphovascular invasion. However, the limited number of our patients did not allow us to determine possible risk factors for extensive DIS.

Complete response was seen in 10 patients in our study. Among the remaining 46 patients, 35 (76.1%) showed no DIS or ≤ 5mm DIS, while the remaining 7 showed 6 to 9 mm DIS. Approximately 93% had < 1 cm DIS. The relatively high rate of DIS ≤ 5mm may be due to thinner macroscopic sections when compared to those used in other studies.

Our landmark in evaluating distal spread was the distal edge of the tumor after NCRT. We were, thus, able to see possible residual tumor foci if the regression was fragmented. However, we did not observe any fragmented tumor regression patterns. One of our most important findings was that tumor regression patterns showed a total shrinkage of the tumor in all patients. This finding should encourage surgeons to accept the distal edge of the postradiation lesion as the landmark for the distal resection margin, which could lead to higher rates of sphincter preservation. Hayden et al. [13] identified microscopic tumor cells outside a visible ulcer in 27 patients (49.1%) who received NCRT. The mean distal scatter was 1 cm, with a maximum of 3 cm in their study, and the authors concluded that the distal margin should not be used to guide the resection margin and a 2 cm margin might not be adequate in those patients. They suggested decisions regarding operations should be based on the preradiation features of the tumors. Discontinuous spread has also been observed in some studies [8,14]. These findings are inconsistent with our results. Although we evaluated the entire distal rectal wall using relatively thinner macroscopic sections in a considerable number of patients, we did not observe any tumor cells scattered outside the tumor. Our results are, though, consistent with more recent studies. Mezhir et al. [15] stated that DIS rarely exceeded 1 cm, although they observed DIS in only 10 of 103 patients, which was lower than our study. They concluded that in patients who received NCRT, local control could be achieved with a distal resection margin of 1 cm [15]. Due to improvements in anastomotic devices and surgical techniques, sphincter preservation in rectal cancer patients has gained more importance. Following recent studies, the 2 cm distal margin tenet has been modified to 1 cm in patients receiving NCRT [6,7,16]. In their systematic review, Pahlman et al. [16] suggested that, for patients receiving preoperative or postoperative radiotherapy, even a ≤ 1 cm distal resection margin was sufficiently disassociated with an increased risk of local recurrence. They also added that their findings supported performing resection with a distal margin shorter than 1 cm when preoperative radiotherapy was selectively used. Similarly, Bujko et al. [6] showed that a distal margin of less than 1 cm was not associated with higher local recurrence and survival in highly selected patients. They also suggested that sphincter preservation might be possible with a distal margin of less than 1 cm in selected patients. However, in none of these studies, the selection criteria were clearly defined.

It has been previously reported that presence of DIS increases the risk of distant metastasis and negatively affects survival [11,17]. This finding is usually the outcome of patients with locally advanced tumors who did not receive preoperative treatment. No relationship has been found between DIS and metastasis or survival in patients who underwent neoadjuvant CRT [1]. In the present study, we found that DIS status does not affect overall survival.

Although none of the patients had a positive distal margin, CRM was positive in three patients, and the distance to CRM was ≤ 1mm in two patients. The survival rates of these five patients were significantly worse than for CRM negative patients.

In patients with mid- and upper-rectum cancers, an adequate distal surgical margin can be easily achieved, and sphincter preservation is possible with an even longer distal margin. For low-lying rectal cancers, a total mesorectal excision is performed, with distal spread being the main concern. Our findings in this study are especially important for this group of patients for whom quality of life is an important issue. The safest approach is a 2 cm distal margin. However, a 1 cm distal margin would seem to be adequate in the vast majority of patients. Identifying patients who are likely to have more than 1 cm DIS requires further studies to avoid unnecessary abdominoperineal resections.

## Figures and Tables

**Table 1:** Modified Ryan’s grading system for tumor regression grading in rectal cancer treated with preoperative therapy [9,10].

Description	Tumor Regression Grade (TRG)
**No viable cancer cells (complete response)**	0
**Single cells or small groups of cancer cells**	1
**Residual cancer with fibrosis**	2
**Extensive residual cancer or minimal response**	3

**Table 2:** The clinicopathological characteristics of the patients.

Characteristic	n: 56
**Age (Year)**	56.96 ± 12.03
**Sex**	
**Male**	18 (%32.1)
**Female**	38 (%67.9)
**BMI (kg/m** **^2^** **)**	26.80 ± 4.42
**Co-morbidity**	
**Yes**	26 (%46.4)
**No**	30 (%53.6)
**ASA Score**	
**I**	21 (%37.5)
**II**	30 (%53.6)
**III**	5 (%8.9)
**Tumor Localization (cm)**	6.46 ± 2.6 (range 2–12)
**Clinical Stage (c Stage)**	
**c T3 N0**	17(%30.4)
**c T3 N1**	36(%64.3)
**c T4 N0**	1 (%1.8)
**c T4 N1**	2 (%3.6)
**Interval Time (day)**	58.7 ± 21.5 (range 24–123)
**Operation Type**	
**Low Anterior Resection (LAR)**	47(%83.9)
**Abdominoperineal Resection (APR)**	9 (%16.1)

**Table 3:** The histopathological outcomes of the patients.

Histopathological Variables	n: 56
**Tumor Size (cm)**	1.98 ± 0.85
**Distal Surgical Margin (Macroscopic) (cm)**	3.15 ± 1.58 (range 0.5–7.2)
**Distal Surgical Margin (Microscopic) (cm)**	2.79 ± 1.54 (range 0.15–6.5)
**Circumferential Resection Margin (cm)**	0.91 ± 0.76
**Circumferential Resection Margin Status**	
**Negative (>1mm)**	51 (%91.1)
**Positive (≤ 1 mm)**	5 (%8.9)
Distal Intramural Spread* **(cm)**	0.56±0.3 (range 0.2–1.8)
**Pathological T Stage**	
**p T0**	10 (%17.8)
**p T1**	3 (%5.4)
**p T2**	12 (%21.4)
**p T3**	30 (%53.6)
**p T4b**	1 (%1.8)
**Harvested Lymph Node**	26.83 ± 8.91(range 12–46)
**Positive Lymph Node**	1.25 ± 2.70 (range 0–11)
**Tumor Regression Grade (TRG)**	
**TRG 0**	10 (%17.9)
**TRG 1**	16 (%28.6)
**TRG 2**	20 (%35.7)
**TRG 3**	9 (%16.1)
**Unknown**	1 (%1.8)
**Perineural Invasion**	
**Yes**	19(%33.9)
**No**	37(%66.1)
**Lymphovascular Invasion**	
**Yes**	22(%39.3)
**No**	31(%55.4)
**Uncertain**	3(%5.4)
**Tumor Stage**	
**Stage 0**	10(%17.9)
**Stage 1**	13(%23.2)
**Stage 2a**	14(%25)
**Stage 3a**	1(%1,8)
**Stage 3b**	15(%26.8)
**Stage 3c**	3(%5.4)

* n: 33
